# Structural characterization of *Platanthera ussuriensis* chloroplast genome and comparative analyses with other species of Orchidaceae

**DOI:** 10.1186/s12864-022-08319-9

**Published:** 2022-01-27

**Authors:** Chenyang Han, Rui Ding, Xiaoyan Zong, Lijie Zhang, Xuhui Chen, Bo Qu

**Affiliations:** 1 College of Bioscience and Biotechnology, Shenyang Agricultural University, Shenyang, 110161 China; 2 College of Land and Environment, Shenyang Agricultural University, Shenyang, 110161 China; 3 College of Forestry, Shenyang Agricultural University, Shenyang, 110161 China

**Keywords:** *Platanthera ussuriensis*, Orchidaceae, Chloroplast genome, Molecular evolution, Phylogenetic analysis, SSR markers, RNA editing site

## Abstract

**Background:**

The genus *Tulotis* has been classified into the genus *Platanthera* in the present taxonomic studies since the morphological characteristics of this genus is very similar to that of *Platanthera*. *Platanthera ussuriensis*, formerly named as *Tulotis ussuriensis*, is a small terrestrial orchid species and has been listed as wild plant under State protection (category II) in China. An improved understanding of the genomic information will enable future applications of conservation strategy as well as phylogenetic studies for this rare orchid species. The objective of this research was to characterize and compare the chloroplast genome of *P. ussuriensis* with other closely related species of Orchidaceae.

**Results:**

The chloroplast genome sequence of *P. ussuriensis* is 155,016 bp in length, which included a pair of inverted repeats (IRs) of 26,548 bp that separated a large single copy (LSC) region of 83,984 bp and a small single copy (SSC) region of 17,936 bp. The annotation contained a total of 132 genes, including 86 protein-coding genes, 38 tRNA genes and 8 rRNA genes. The simple sequence repeat (SSR) analysis showed that there were 104 SSRs in the chloroplast genome of *P. ussuriensis*. RNA editing sites recognition indicated 72 RNA editing events occurred, and all codon changes were C to T conversions. Comparative genomics showed that the chloroplast sequence of *Platanthera* related species were relatively conserved, while there were still some high variation regions that could be used as molecular markers. Moreover, *Platanthera* related species showed similar IR/SSC and IR/LSC borders. The phylogenetic analysis suggested that *P. ussuriensis* had a closer evolutionary relationship with *P. japonica* followed by the remaining *Platanthera* species.

**Conclusion:**

Orchidaceae is a key group of biodiversity protection and also a hot spot group in the plant taxonomy and evolution studies due to their characteristics of high specialization and rapid evolution. This research determined the complete chloroplast genome of *P. ussuriensis* for the first time, and compared the sequence with other closely related orchid species. These results provide a foundation for future genomic and molecular evolution of the Orchidaceae species, and provide insights into the development of conservation strategy for *Platanthera* species.

**Supplementary Information:**

The online version contains supplementary material available at 10.1186/s12864-022-08319-9.

## Background

Orchidaceae is one of the largest family of angiosperms and also a key group of biodiversity protection [1, 2]. The group of orchid species are widely distributed in almost all kinds of terrestrial ecosystems in the world, and is a hot spot group in the plant taxonomy and evolution studies due to the characteristics of high specialization and rapid evolution [3]. The classification system of orchid species was mainly based on morphological characteristics for a long time [4]. However, it is now widely recognized that the identification of orchid species is difficult, especially in the non-flowering period when many orchid species plants have very similar morphological characteristics. On the other hand, orchid flowers could be influenced by the selection pressure of their pollinators so easily that it is still difficult to distinguish them even at flowering stages. Moreover, many orchid species could crossbreed successfully in a wide range, resulting in many intermediate types and large natural variations. All of these make it very difficult for traditional taxonomy based on the morphological characteristics of orchids [5–9]. In terms of phylogeny, it is also difficult to understand the phylogenetic relationship between various groups because of the relatively complex evolutionary process and multi lineage origin of many orchid genera [10, 11].

As the photosynthetic organ in plants, chloroplast has an independent genome and the capacity of replication, transcription and translation [12]. The chloroplast genome of angiosperms usually consists of four parts and form a circular double chain structure including one large single copy (LSC), one small single copy (SSC), and two inverted repeats (IRs) [13]. It is an ideal tool for studying genetic differences and phylogenetic relationships among species due to its small length, high conservation, large number of gene copies and rapid evolution rate [14–16]. In recent years, with more and more chloroplast genomes being sequenced, the research on plant phylogeny based on chloroplast genome is increasing which provides an effective solution for systematic problems of some difficult taxa [17, 18].

At present, with the progress of genome sequencing technology and the increasing of genomics research, the taxonomic research of orchids has entered a stage of adjustment [19–23]. Therefore, the taxonomy system and phylogenetic status of orchids have been changed a lot, such as the *Tulotis* genus, which has been classified into the genus *Platanthera* in the present taxonomic studies since the morphological characteristics of this genus is very similar to that of *Platanthera*.

*Platanthera ussuriensis* (Regel) Maxim, formerly named as *Tulotis ussuriensis*, is a small terrestrial orchid species mainly distributed in China, Korean, Russia and Japan. Due to the increasing of habitat fragmentation and human disturbance, the survival of this species has been seriously threatened nowadays, and it has been listed as wild plant under State protection (category II) in China. In the present study, we sequenced the complete chloroplast genome of *P. ussuriensis*, and compared the resulting genome sequence with six other published chloroplast genomes of closed related species. Our aims were: (1) to investigate structural pattern of whole chloroplast genome including genome structure, gene order, and gene content; (2) to perform genome-based analysis and compare the differences among selected closely related species; (3) to reconstruct a chloroplast phylogeny for *Platanthera* orchids species using their whole chloroplast sequences. Our results would enrich the chloroplast genome database of *Platanthera* species, and get an insight into the evolutionary history of Orchidaceae family.

## Results

### General feature of the chloroplast genome

**Fig. 1:**
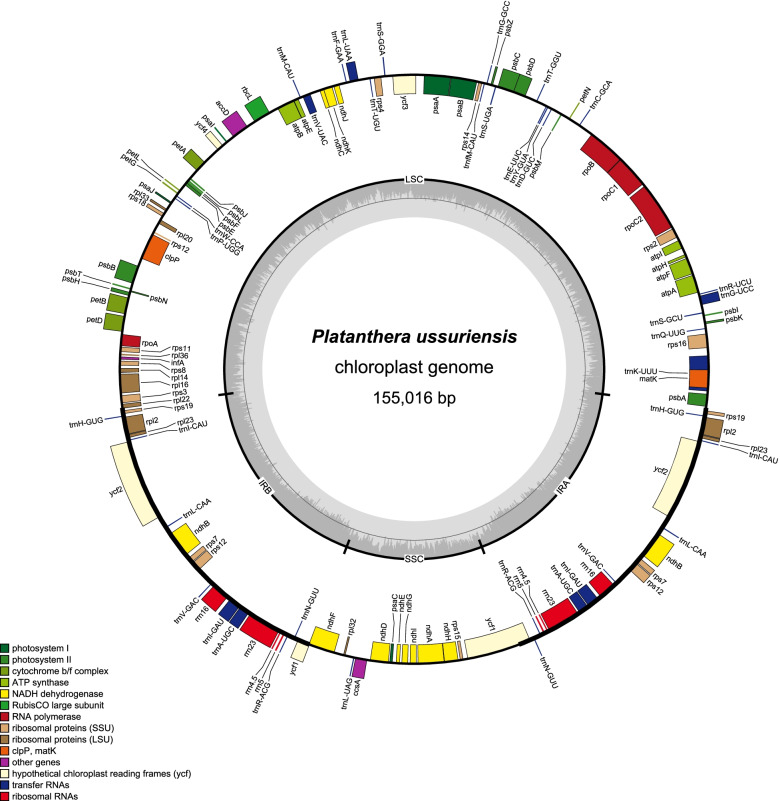
Chloroplast genome map of *Platanthera ussuriensis*. Genes drawn outside the outer circle are transcribed clockwise, and those inside are transcribed counter-clockwise. Genes belonging to different functional groups are color-coded. The dark gray in the inner circle indicates GC content of the chloroplast genomes

The chloroplast genome of *P. ussuriensis* is a circular molecule of 155,016 bp (Fig. 1), consisting of a large single copy (LSC) region of 83,984 bp, a small single copy (SSC) region of 17,936 bp, and a pair of inverted repeats (IRa and IRb) of 26,548 bp. The overall GC content was 36.7%. The GC contents were unevenly distributed across different regions of the cp genome, which were found to be 34.2%, 29.3%, and 43.1% for the LSC, SSC, and IR regions, respectively (Table 1). The chloroplast genome contained 132 genes, including 86 protein-coding genes, 38 tRNA genes and 8 rRNA genes. Among them, 92 genes were unique, and 20 genes were duplicated in the IR regions (4 rRNA genes, 8 tRNA genes and 8 protein-coding genes). There were 22 intron-containing genes including 14 protein-coding genes and 8 tRNA genes, and 3 genes (*rps12*, *ycf3* and *clpP*) possess two introns. In particular, 5′-end and 3′-end exons of *rps12* gene were respectively located in the LSC and IR regions (Table 2).

**Table 1:** Comparative analysis of chloroplast genomes of four *Platanthera* species

Characteristics		*Platanthera ussuriensis*	*Platanthera japonica*	*Platanthera chlorantha*	*Platanthera mandarinorum*
GenBank accession No.		MN686021	MN631092	MK937914	MN200370
Size (base pair, bp)		155,016	155,409	154,260	154,162
LSC length (bp)		83,984	84,049	83,279	83,325
SSC length (bp)		17,936	17,494	17,759	17,757
IR length (bp)		26,548	26,933	26,611	26,540
Number of genes		132	133	134	132
Protein-coding genes		86	87	88	86
tRNA genes		38	38	38	38
rRNA genes		8	8	8	8
GC content	Total (%)	36.7	36.9	36.7	36.9
	LSC (%)	34.2	34.4	34.3	34.3
	SSC (%)	29.3	29.7	29.4	29.5
	IR (%)	43.1	43.2	43.1	43.1

**Table 2:** Chloroplast gene content and functional classification in *Platanthera ussuriensis*

Group of gene	Name of gene	Number
Photosystem I	*psaB*, *psaA*, *psaI*, *psaJ*, *psaC*	5
Photosystem II	*psbA*, *psbK*, *psbI*, *psbM*, *psbD*, *psbC*, *psbZ*, *psbJ*, *psbL*, *psbF*, *psbE*, *psbB*, *psbT*, *psbN*, *psbH*	15
Cytochrome b/f complex	*petN*, *petA*, *petL*, *petG*, *petB*^a^, *petD*^a^	6
ATP synthase	*atpA*, *atpF*^a^, *atpH*, *atpI*, *atpE*, *atpB*	6
NADH dehydrogenase	*ndhJ*, *ndhK*, *ndhC*, *ndhB*^a,c^, *ndhF*, *ndhD*, *ndhE*, *ndhG*, *ndhI*, *ndhA*^a^, *ndhH*	12
RubisCO large subunit	*rbcL*	1
RNA polymerase	*rpoC2*, *rpoC1*^a^, *rpoB*, *rpoA*	4
Ribosomal proteins (SSU)	*rps12*^b,c^, *rps16*^a^, *rps2*, *rps14*, *rps4*, *rps18*, *rps11*, *rps8*, *rps3*, *rps19*^c^, *rps7*^c^, *rps15*	15
Ribosomal proteins (LSU)	*rpl33*, *rpl20*, *rpl36*, *rpl14*, *rpl16*, *rpl22*, *rpl2*^a,c^, *rpl23*^c^, *rpl32*	11
Other genes	*accD*, *infA*, *ccsA*, *clpP*^b^, *matK*	5
Hypothetical chloroplast reading frames (*ycf*)	*ycf3*^b^, *ycf4*, *ycf2*^c^, *ycf1*^c^	6
Transfer RNAs	*trnK-UUU*^a^, *trnQ-UUG*, *trnS-GCU*, *trnG-UCC*^a^, *trnR-UCU*, *trnC-GCA*, *trnD-GUC*, *trnY-GUA*, *trnE-UUC*, *trnT-GGU*, *trnS-UGA*, *trnG-GCC*, *trnfM-CAU*, *trnS-GGA*, *trnT-UGU*, *trnL-UAA*^a^, *trnF-GAA*, *trnV-UAC*^a^, *trnM-CAU*, *trnW-CCA*, *trnP-UGG*, *trnH-GUG*^c^, *trnI-CAU*^c^, *trnL-CAA*^c^, *trnV-GAC*^c^, *trnI-GAU*^a,c^, *trnA-UGC*^a,c^, *trnR-ACG*^c^, *trnN-GUU*^c^, *trnL-UAG*	38
Ribosomal RNAs	*rrn16*^c^, *rrn23*^c^, *rrn4.5*^c^, *rrn5*^c^	8
Total		132

^a^Gene with one intron; ^b^Gene with two introns; ^c^Gene with two copies

The amino acid frequency and codon usage were analyzed based on the 86 sequences of protein-coding genes which encoded a total of 26,587 codons. The results showed that leucine (10.32%) was the most abundant amino acid, whereas cysteine (1.21%) was the least abundance. The most and least used codons were AUU (1147) encoding lsoleucine, and UGC (78) encoding cysteine, respectively. Furthermore, the highest RSCU value was for UUA (1.94) in leucine, and the lowest was AGC (0.34) in serine and GAC (0.34) in aspartic acid, while methionine and tryptophan showed RSCU equals to 1 (Supplemental Table 1).

### Repeat and SSR analysis

**Fig. 2:**
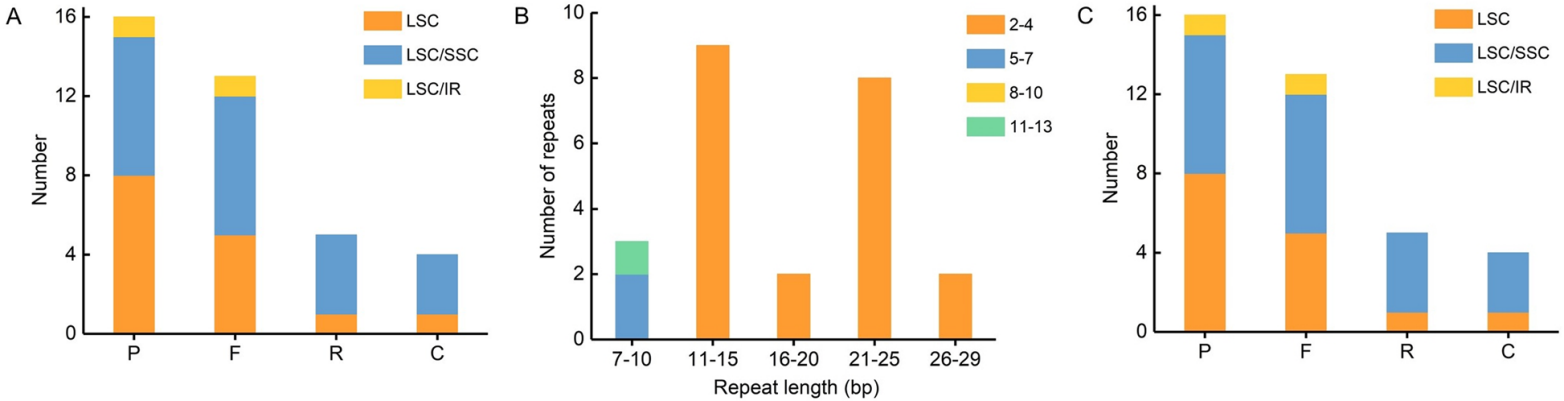
Analysis of repeated sequences in the *Platanthera ussuriensis* chloroplast genome. **A** Frequency and locations of four repeat types, **B** Frequency of tandem repeats by length and their copy number, **C** Frequency of tandem repeats by their region and location

REPuter analysis revealed 38 oligonucleotide repeats (> 30 bp) from which 16 were palindromic (P), 13 were forward (F), 5 were reverse (R), and 4 were complement (C). The repeat ranged from 30 to 39 bp in length, and their repeat number varied from 1 to 4. A total of 15 oligonucleotide repeats were identified in LSC region, 23 were distributed in junctions between the regions among which 21 were present in LSC/SSC region, and 2 were present in LSC/IR region (Supplemental Table 2, Fig. 2A). In addition, a total of 24 tandem repeats were identified (Supplemental Table 3). The length of these repeats varied from 7 to 29 bp, and their copy number ranged from 2 to 13 (Fig. 2B). Tandem repeats were distributed in all the LSC, SSC and IRs regions, and 15, 6, and 3 were present in IGS, intron and CDS region, respectively (Fig. 2C).

**Fig. 3:**
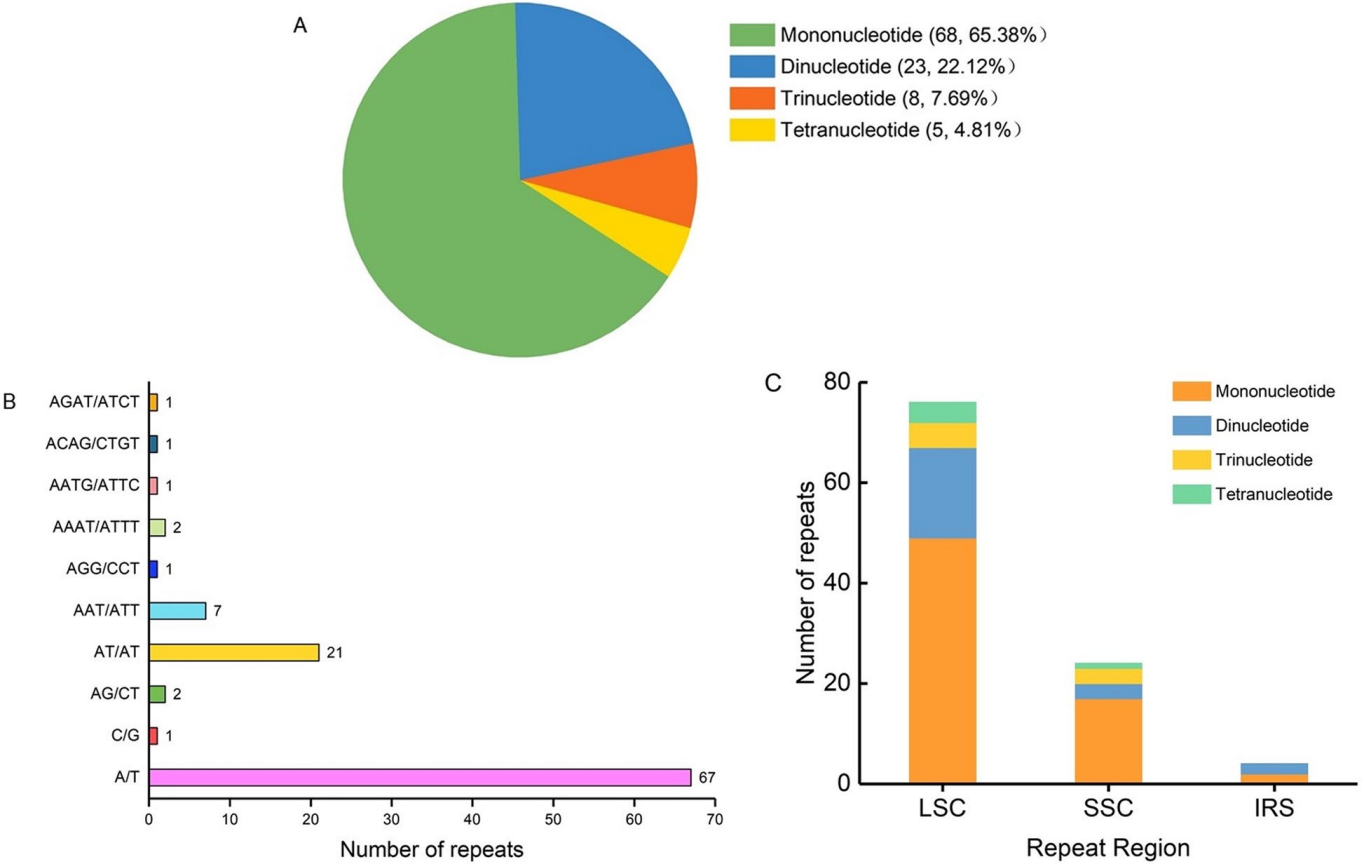
Analysis of SSRs in the *Platanthera ussuriensis* chloroplast genome. **A** Number and frequency of different SSR types, **B** Number of different SSR motifs, **C** Types and frequency of SSRs in different regions

A total of 104 SSRs were detected (Supplemental Table 4). Among these SSRs, there were 68, 23, 8 and 5 for mono-, di-, tri- and tetra- nucleotide repeats, respectively, and no pentanucleotide and hexanucleotide repeats were found (Fig. 3A). The majority of the mononucleotides were composed of A/T, and most of the dinucleotides were AT/AT (Fig. 3B). There were 76, 24, and 4 SSR repeats distributed in LSC, SSC, and IR region, respectively (Supplemental Table 5, Fig. 3C).

### Recognition of RNA editing sites

**Fig. 4:**
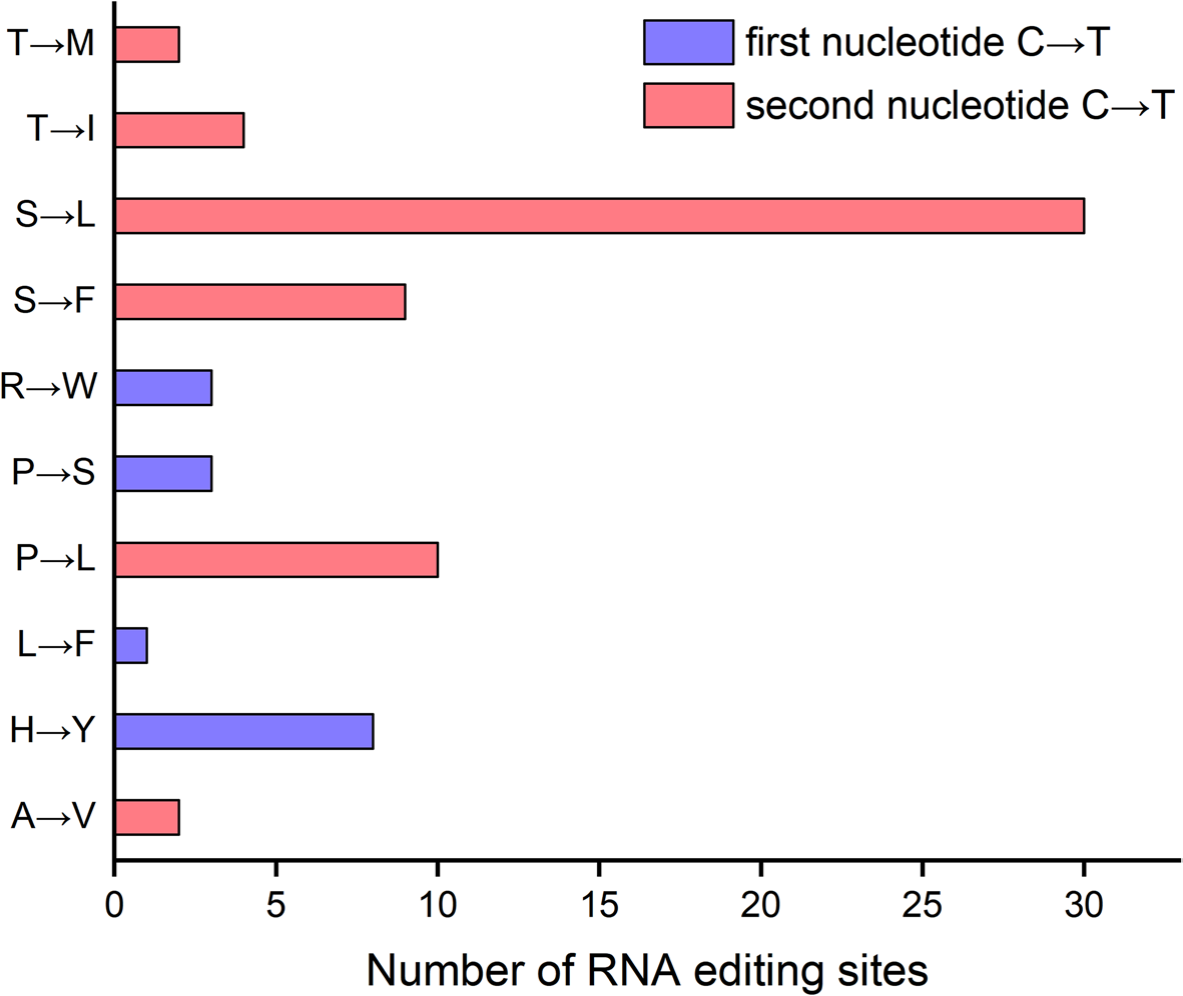
Number of RNA editing sites by different amino acid changes and C to T location of the codons

PREP software found 35 genes predicting RNA editing sites in the chloroplast genome of *P. ussuriensis* among which 10 genes (*petD*, *petG*, *petL*, *psaB*, *psbB*, *psbE*, *psbL*, *rpl2*, *rpl23*, *rps2*) had been identified no sites (Supplemental Table 6). In the rest 25 protein-coding genes, a total of 72 RNA editing sites were predicted. Among these genes, the largest number of RNA editing sites were identified in the *ndhB* gene (11 sites), following by *ndhD*, *ndhF*, *rpoB*, and *rpoC1*, with 6 sites respectively. All codon changes were C to T conversions, and 15 editing sites were the first nucleotide of the codon, while 57 editing sites were the second nucleotide of the codon. The most abundant amino acid change was S to L, with 30 out of 72 editing events, and the rarest was L to F, with only one out of all editing events (Fig. 4).

### IR expansion and contraction

**Fig. 5:**
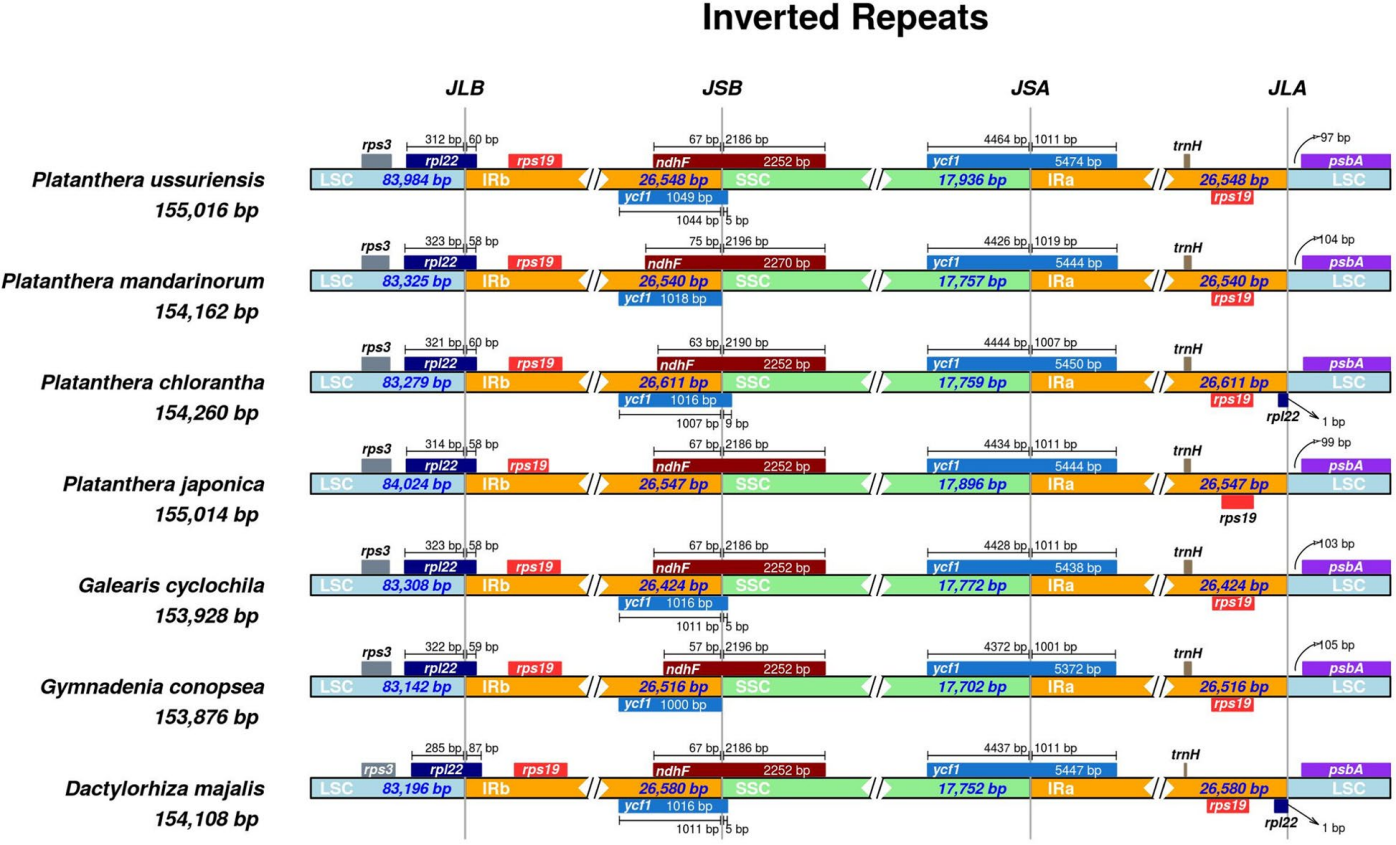
Comparison of the borders of LSC, SSC and IR regions among seven sequenced Orchidaceae chloroplast genomes. Genes transcribed forward are shown above the lines whereas genes transcribed reversely are shown below the lines. Gene lengths in the corresponding regions are displayed above the boxes of gene names. The number of bp represented by the arrow show genes away from specific region of chloroplast genome. JLB (LSC/IRb), JSB (IRb/SSC), JSA (SSC/IRa), and JLA (IRa/LSC) denote the junction sites between each corresponding two region on the chloroplast genome

Comprehensive comparison at the LSC/IRs/SSC boundaries was analyzed among *P. ussuriensis* and six other closely related orchid species (Fig. 5). Although the length of the IR regions that ranged from 26,424 bp to 26,933 bp, varied little among the seven species, some differences in the IR expansions and contractions were observed. These chloroplast genomes showed similar SSC/IR borders which were located in the coding region of the *ndhF* (IRb/SSC) and *ycf1* (SSC/IRa) gene, respectively. The IRb region extended into the *rpl22* gene in all these chloroplast genomes, with the length ranging from 58 to 87 bp. The *rpl22* gene was duplicated only in *Dactylorhiza majalis* and *P. chlorantha* which located in the IRa/LSC border while not in the other five species. The *psbA* gene was included in the LSC region in all these species, 97-111 bp away from the IRa/LSC border. In addition, the *trnH* and *rps19* gene cluster were also totally located within the IR region and duplicated. The *ycf1* pseudogene was existed in all of these chloroplast genomes at the IRb/SSC border except for *P. japonica*, and an overlap was existed between *ycf1* pseudogene and *ndhF* gene.

### Comparison of chloroplast genome sequences

**Fig. 6:**
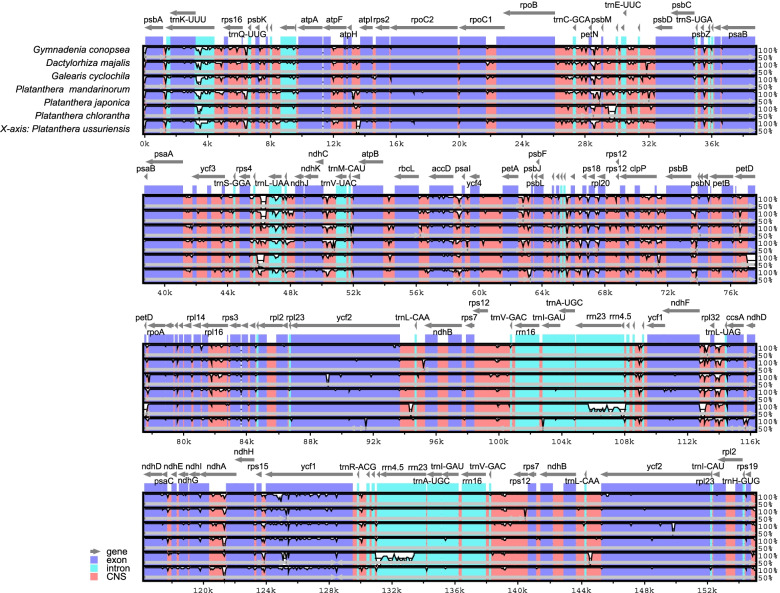
Sequence alignment of seven Orchidaceae chloroplast genomes using mVISTA. The vertical scale indicates the percentage of identity, ranging from 50 to 100%. The horizontal axis indicated the coordinates within the chloroplast genome. Genome regions are color coded as exton, intron, and conserved non-coding sequences (CNS)

The chloroplast genome sequences were compared among *P. ussuriensis* and six other closely related orchid species (Fig. 6). Overall, the LSC and SSC regions in these chloroplast genomes were more divergent than the IR regions. Non-coding regions presented higher divergence than coding regions, but the rRNA genes of *P. japonica* had higher variability regions when comparing with the other six species. The highest polymorphic regions were located in the intergenic regions, such as *trnK-UUU*—*matK*, *ndhF*—*rpl32*, and *rpl32*—*trnL-UAG*.

### Phylogenetic analysis

**Fig. 7:**
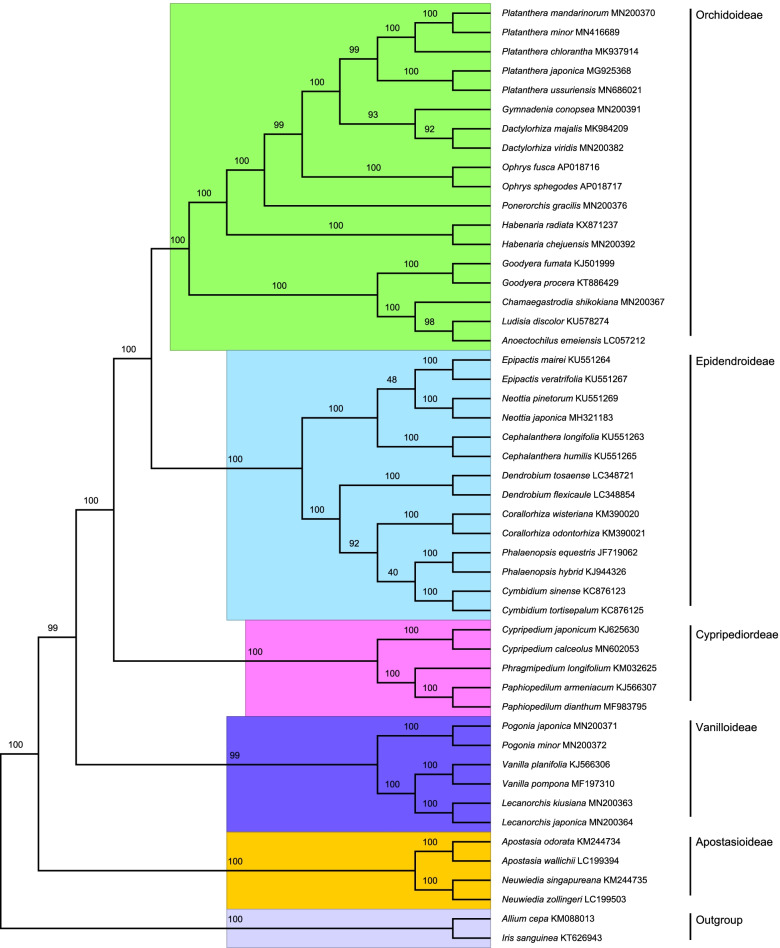
Phylogenetic tree reconstruction of Orchidaceae using maximum likelihood (ML) based on whole chloroplast genome sequences, with *Allium cepa* and *Iris sanguinea* as the outgroup. The branch support was determined by computing 1000 non-parametric bootstrap replicates

The overall topology of the phylogenetic trees produced with Maximum-Likelihood (ML) and Neighbor-Joining (NJ) methods were similar based on our whole chloroplast genome sequences. The ML tree (Fig. 7) showed that all species formed five major clades which correspond to the five subfamilies of Orchidaceae. Within the *Platanthera* genus, all the *Platanthera* species were clustered in a single major clade, and *P. ussuriensis* and *P. japonica* were located in adjacent branches followed by the remaining *Platanthera* species.

## Discussion

In this study, we reported the complete chloroplast genome of *P. ussuriensis* for the first time and compared with other six species of Orchidaceae in order to broaden the knowledge about the genome organization and molecular evolution of the Orchidaceae species.

The chloroplast genomes of angiosperms have conserved features and almost similar gene content and organization within and among different plant lineage [24, 25]. The obtained chloroplast genome of *P. ussuriensis* in this study had typical characteristics of angiosperm chloroplast genomes, and the general feature were not significantly different from those of its closely related species [10, 26, 27]. However, the GC contents of these orchid species in IR region were remarkably higher than that in other two regions, and this phenomenon was also common in other plant species [28, 29]. The reason for this phenomenon is generally believed to be the existence of rRNA gene and tRNA gene within the region [30–32]. Although GC content of chloroplast genome varies greatly among different plant species [16, 17, 20, 22, 31–33], the GC content of different species of *Platanthera* genus in our study were very close.

Simple sequence repeats (SSRs), known as microsatellites, are very often used as genetic molecular markers in population genetic relationship and phylogenetic studies because of their widely distribution and highly polymorphism at the intraspecific level [15, 34–41]. In this study, more than 100 SSRs were found in the *P. ussuriensis* chloroplast genome. Most of them were AT rich, and the mononucleotides were found to be the most abundant repeats. Similar results have been found in other plant species, which were consistent with the contention that chloroplast SSRs are generally composed by short polyA or polyT repeats and the mononucleotide repeats tend to be the most frequent types of chloroplast SSRs [42, 43]. The oligonucleotide repeats found in this study could also be helpful for identification of polymorphic regions within *P. ussuriensis* and other Orchidaceae species.

RNA editing is one of the most important method to regulate gene expression in higher plants after transcription [44–46]. Of the 72 RNA editing sites of *P. ussuriensis*, 79.17% occurred at the second codons, and the rest occurred at the first codons. This phenomenon was consistent with the results of RNA editing in earlier studies [47, 48]. It has been reported that most RNA editing tends to shift amino acids from polar to nonpolar and from hydrophilic to hydrophobic, which results in increased hydrophobicity of proteins [49–51]. In our present study, 61 of the 72 RNA editing sites were eventually converted to nonpolar and hydrophobic amino acids, including valine (V), leucine (L), isoleucine (I), methionine (M), tryptophan (W) and phenylalanine (F). These results could provide data for the evolutionary investigations of the genus *Platanthera*, and the same was true for the analysis of codon usage frequency and relative synonymous codon usage (RSCU). In addition, codon usage was biased towards A and U at the third codon position, which is similar to the trend observed in most angiosperm chloroplast genomes [52].

Although the IR region of chloroplast genome is considered to be the most conservative region, the contraction and expansion of its borders are common events in the evolution of chloroplast genome, and therefore the main reason for the variation of chloroplast genome length [53–55]. In eudicots, the *trnH* gene is usually located in the LSC region and the borders of the IR regions are found to contain the *rps19* or *rps19* pseudogene [56–59], while IRb/LSC is located downstream of the *psbA* gene and IRs have expanded to include *trnH* and *rps19* gene cluster in most monocots [60, 61]. However, the study of IR junctions between LSC and SSC showed remarkable changes. Our results showed that the *trnH* and *rps19* gene cluster was included in the IR region of the orchid species chloroplast genome, which was consistent with its location in most monocot genomes. However, the IR expanded into the *rpl22* gene in all of these orchid species and created a pseudogene at the IRa/LSC border in *D. majalis* and *P. chlorantha* species. Similar results were also found in other orchid species [62] while was significantly different from that in other monocot lineages [24, 61]. The fluxes in the IR boundaries have been suggested to implicate the taxonomic relationships among angiosperms, and our results revealed that the expansion was unusual in Orchidaceae species and the degrees of IR to LSC expansions were much larger than other monocot lineages. Variation of expansions has also been detected at IR/SSC boundaries of monocot chloroplast genomes, and there was an IR contraction existed in *Phalaenopsis* chloroplast genome in which the *ycfl* gene was completely included in SSC region [62]. In our study, the *ycf1* gene spanned the SSC/IRa boundary in *Platanthera* and adjacent species, indicating that the degrees of expansion may varied greatly among different orchid genus. These differences would be useful for studying the taxonomy and evolutionary relationships of different species.

ML and NJ analysis were used to construct the evolutionary tree, and we found that ML and NJ trees had similar phylogenetic topologies with five major clades (Orchidoideae, Epidendroideae, Cypripedioideae, Vanilloideae, and Apostasioideae). The *Platanthera* branch had a strong supported topology and showed that *P. ussuriensis* and *P. japonica* have a closer evolutionary relationship. This phylogenetic topology was highly consistent with previously published phylogenetic analysis based on complete plastid genomes [10] and study based on several genes from Orchidaceae [63]. The data we presented here and in conjunction with previously published chloroplast genome sequences would help to expand our understanding of the evolutionary history of the orchid species, especially for the systematics and evolution of the *Platanthera* genus.

## Conclusion

*Platanthera ussuriensis*, formerly named as *Tulotis ussuriensis*, is a small terrestrial orchid species and has been listed as wild plant under State protection (category II) in China. A great understanding of genomic information provides insights into the conservation works of *Platanthera* species. This research focused on the chloroplast sequence analysis of *P. ussuriensis* species, including general feature, SSR sites, RNA editing sites, and comparison with other closely related species. The results provide a guide for future genomic and conservation strategy of the Orchidaceae species.

## Methods

### Ethical statement

No specific permits were required for the collection of specimens for this study. This research was carried out in compliance with the relevant laws of China.

### Plant materials and chloroplast genome sequencing

Fresh leaves from an adult *P. ussuriensis* plant were collected and identified by Dr. Bo Qu and Dr. Xuhui Chen of Shenyang Agricultural University from Daqinggou nature reserve, Fuxin, northeast China. Total genomic DNA was extracted using the CTAB method [64], and the specimen was stored with the archival number of ORCHID_PLA_USS_01 at College of Bioscience and Biotechnology of Shenyang Agricultural University. Next-generation sequencing was performed with an Illumina Miseq sequencing platform by Shanghai Personal Biotechnology Co. Ltd, China. The paired-end library was constructed using TruSeqTM DNA Sample Prep Kit, and then paired reads with 400 bp insert size were sequenced and yielded at least 2 GB clean data. The qualities of the clean reads were checked using FastQC v0.11.7.

### Chloroplast genome assembly and annotation

For de novo chloroplast genome assembly, all the paired-end sequences were assembled into contigs, and neighboring contigs with paired-end support for continuity were merged into scaffolds using SPAdes v.3.11.0 software. Then, using the published chloroplast genome and protein coding gene sequences of related species as reference, the scaffolds with gene matching were picked out and assembled into the chloroplast genome with Velvet v.1.2.07 [65]. The online GeSeq-Annotation of Organellar Genomes program coupled with manual corrections for start and stop codons were used to annotate the complete chloroplast genome [66]. The tRNA genes were identified using GeSeq and tRNAscan-SE v2.0.5 [67]. A circular genome map was drawn using the OGDRAW program (https://chlorobox.mpimp-golm.mpg.de/OGDraw) [68].

### Sequence analysis and statistics

The web-based software REPuter (https://bibiserv.cebitec.uni-bielefeld.de/reputer/) [69] was used to analyze the repeat sequences, which included forward, reverse, complement and palindromic repeats with minimal repeat size set to 30 bp, hamming distance set to 3 and maximum computed repeats set to 90. In addition, the tandem repeat sequences were detected by Tandem Repeats Finder with default parameters (http://tandem.bu.edu/trf/trf.html) [70]. Simple sequence repeats(SSR) which is a tract of repetitive DNA and usually ranges in length from 1 to 6 nucleotides were detected via MISA (https://webblast.ipk-gatersleben.de/misa/) by setting the minimum number of repeats to 10, 5, 4, 3, 3 and 3 for mononucleotide, dinucleotide, trinucleotide, tetranucleotide, pentanucleotide and hexanucleotide, respectively [71].

Condon usage was analyzed by MEGA-X software (version 10.1.6) [72], and the relative synonymous codon usage (RSCU) and amino acid frequencies were calculated with default settings [73]. The putative RNA editing sites of 86 protein-coding genes in *P. ussuriensis* were predicted by the online PREP-Cp program (http://prep.unl.edu/) with default parameters, and the cutoff value was set to 0.8 to ensure prediction accuracy [74].

### Sequence divergence and genome comparison

The mVISTA program in LAGAN mode [75] was used to make pairwise alignments and sequence divergence within *P. ussuriensis* and six other closely related species, i.e., *P. chlorantha*, *P. japonica*, *P. mandarinorum*, *Galearis cyclochila*, *D. majalis* and *Gymnadenia conopsea*. The result was plotted using the mVISTA tools (http://genome.lbl.gov/vista/mvista/submit.shtml) with *P. ussuriensis* as a reference [76]. The contraction and expansion of the IR boundaries between the four main regions (LSC/IRa/SSC/IRb) of the seven chloroplast genome sequences were visualized using the online software IRSCOPE (https://irscope.shinyapps.io/irapp/) [77].

### Phylogenetic analysis

To determine the location of *P. ussuriensis* in Orchidaceae family and to analyze the phylogenetic relationship of the *Platanthera* genus, a total of 47 chloroplast genomes from the family Orchidaceae were selected to construct phylogenetic trees, with two species *Allium cepa* and *Iris sanguinea* selected as the outgroup. We downloaded chloroplast genome sequences of 46 species across Orchidaceae family which correspond to all five subfamilies of Orchidaceae (Orchidoideae, Epidendroideae, Cypripediordeae, Vanilloideae, and Apostasioideae), selecting two chloroplast genomes per genus except where there was only one representative species in any genus which was also included in the analyses. These sequences were aligned using HomBlocks [78] with the Gblocks method [79], resulting in 9,262 aligned characters. Maximum-Likelihood (ML) and Neighbor-Joining (NJ) analyses were performed. ML tree was built using PhyML 3.0 [80] with a General Time Reversible + Proportion Invariant + Gamma (GTR + I + G) model. NJ tree was bulit under Maximum Composite Likelihood method with Gamma distribution rates (parameter = 4) using MEGA6 [81]. 1000 non-parametric bootstrap replicates were performed to estimate the support of the data for each internal branch of the phylogeny.

## Supplementary Information

**Additional file 1.**

## Data Availability

The datasets generated or analyzed during the current study are available in the NCBI Bioproject repository MN686021, (https://www.ncbi.nlm.nih.gov/nuccore/MN686021.1).

## Abbreviations

C: Complement
CDS: Coding sequence
CNS: Conserved non-coding sequences
F: Forward
IGS: Intergenic spacer
IRs: Inverted repeats
LSC: Large single copy
ML: Maximum-Likelihood
NJ: Neighbor-Joining
P: Palindromic
R: Reverse
RSCU: Relative synonymous codon usage
SSC: Small single copy
SSR: Simple sequence repeat
