# Supplementary material for: Structural characterization of Platanthera ussuriensis chloroplast genome and comparative analyses with other species of Orchidaceae
Source: BMC Genomics. 2022 Jan 27;23:84. doi: 10.1186/s12864-022-08319-9 (PMC8796522; doi:10.1186/s12864-022-08319-9)
Supplement: Supplementary file 1 — Additional file 1. [file 12864_2022_8319_MOESM1_ESM.docx]

**Additional file**

Table S1 Codon usage and amino acid frequencies of *Platanthera ussuriensis.*

Table S2 Oligonucleotide repeats (>30 bp) identified within the chloroplast genome of *Platanthera ussuriensis.*

Table S3 Tandem repeat sequences in the *Platanthera ussuriensis* chloroplast genome.

Table S4 Total number of SSRs identified within the chloroplast genome of *Platanthera ussuriensis*.

Table S5 SSRs within the *Platanthera ussuriensis* chloroplast genome.

Table S6 RNA editing sites and amino acid changes in the chloroplast genome of *Platanthera ussuriensis*.

Table S1 Codon usage and amino acid frequencies of *Platanthera ussuriensis*.

| Amino acid | Codons | Number | RSCU | Amino acid frequencies | Amino acid | Codons | Number | RSCU | Amino acid frequencies |
| --- | --- | --- | --- | --- | --- | --- | --- | --- | --- |
| Phe | UUU | 1011.0 | 1.29 | 5.93 | Tyr | UAU | 791.0 | 1.61 | 3.71 |
|  | UUC | 560.0 | 0.71 |  |  | UAC | 193.0 | 0.39 |  |
| Leu | UUA | 882.0 | 1.94 | 10.32 | Cys | UGU | 242.0 | 1.51 | 1.21 |
|  | UUG | 549.0 | 1.20 |  |  | UGC | 78.0 | 0.49 |  |
|  | CUU | 574.0 | 1.26 |  | Trp | UGG | 458.0 | 1.00 | 1.73 |
|  | CUC | 196.0 | 0.43 |  | His | CAU | 487.0 | 1.54 | 2.39 |
|  | CUA | 350.0 | 0.77 |  |  | CAC | 146.0 | 0.46 |  |
|  | CUG | 183.0 | 0.40 |  | Gln | CAA | 695.0 | 1.51 | 3.47 |
| Ile | AUU | 1147.0 | 1.48 | 8.77 |  | CAG | 225.0 | 0.49 |  |
|  | AUC | 420.0 | 0.54 |  | Asn | AAU | 993.0 | 1.56 | 4.79 |
|  | AUA | 758.0 | 0.98 |  |  | AAC | 277.0 | 0.44 |  |
| Met | AUG | 671.0 | 1.00 | 2.53 | Lys | AAA | 1096.0 | 1.48 | 5.59 |
| Val | GUU | 505.0 | 1.46 | 5.23 |  | AAG | 386.0 | 0.52 |  |
|  | GUC | 187.0 | 0.54 |  | Asp | GAU | 892.0 | 1.66 | 4.06 |
|  | GUA | 483.0 | 1.39 |  |  | GAC | 183.0 | 0.34 |  |
|  | GUG | 212.0 | 0.61 |  | Glu | GAA | 1052.0 | 1.48 | 5.36 |
| Gly | GGU | 554.0 | 1.25 | 6.69 |  | GAG | 369.0 | 0.52 |  |
|  | GGC | 195.0 | 0.44 |  | Ser | AGU | 406.0 | 1.18 | 7.81 |
|  | GGA | 712.0 | 1.61 |  |  | AGC | 116.0 | 0.34 |  |
|  | GGG | 311.0 | 0.70 |  |  | UCU | 618.0 | 1.79 |  |
| Pro | CCU | 425.0 | 1.55 | 4.05 |  | UCC | 326.0 | 0.95 |  |
|  | CCC | 229.0 | 0.85 |  |  | UCA | 427.0 | 1.24 |  |
|  | CCA | 308.0 | 1.15 |  |  | UCG | 176.0 | 0.51 |  |
|  | CCG | 121.0 | 0.45 |  | Arg | AGA | 505.0 | 1.88 | 6.08 |
| Thr | ACU | 540.0 | 1.59 | 5.13 |  | AGG | 180.0 | 0.67 |  |
|  | ACC | 247.0 | 0.73 |  |  | CGU | 347.0 | 1.29 |  |
|  | ACA | 425.0 | 1.25 |  |  | CGC | 106.0 | 0.39 |  |
|  | ACG | 147.0 | 0.43 |  |  | CGA | 351.0 | 1.31 |  |
| Ala | GCU | 624.0 | 1.83 | 5.14 |  | CGG | 122.0 | 0.45 |  |
|  | GCC | 197.0 | 0.58 |  | End | UAA | 44.0 | 1.45 | None |
|  | GCA | 403.0 | 1.18 |  |  | UAG | 26.0 | 0.86 |  |
|  | GCG | 137.0 | 0.40 |  |  | UGA | 21.0 | 0.69 |  |

Table S2 Oligonucleotide repeats (>30 bp) identified within the chloroplast genome of *Platanthera ussuriensis.*

| Repeat length of the first part | Starting position of the first part | Repeat length of the second part | Starting position of the second part | Repeat number | Match direction | Location |
| --- | --- | --- | --- | --- | --- | --- |
| 30 | 28436 | 30 | 50354 | 1 | C | LSC |
| 33 | 1296 | 33 | 121330 | 1 | C | LSC/SSC |
| 30 | 1314 | 30 | 113089 | 1 | C | LSC/SSC |
| 31 | 66934 | 31 | 113094 | 1 | C | LSC/SSC |
| 30 | 9408 | 30 | 35843 | 1 | F | LSC |
| 30 | 38048 | 30 | 40272 | 1 | F | LSC |
| 30 | 46085 | 30 | 50358 | 1 | F | LSC |
| 31 | 46089 | 31 | 50360 | 1 | F | LSC |
| 30 | 1291 | 30 | 1293/121324/121322/121326 | 4 | F | LSC,LSC/SSC |
| 39 | 43018 | 39 | 99289 | 1 | F | LSC/IR |
| 31 | 1315 | 31 | 113091 | 1 | F | LSC/SSC |
| 31 | 3531 | 31 | 121332 | 1 | F | LSC/SSC |
| 30 | 3533 | 30 | 113099 | 1 | F | LSC/SSC |
| 31 | 66925 | 31 | 121332 | 1 | F | LSC/SSC |
| 33 | 1297 | 33 | 66934 | 1 | P | LSC |
| 30 | 1305 | 30 | 3533 | 1 | P | LSC |
| 32 | 5870 | 32 | 66928 | 1 | P | LSC |
| 34 | 7955 | 34 | 44462 | 1 | P | LSC |
| 31 | 28432 | 31 | 50357 | 1 | P | LSC |
| 33 | 35009 | 33 | 44465 | 1 | P | LSC |
| 30 | 46080 | 30 | 69414 | 1 | P | LSC |
| 30 | 50359 | 30 | 73626 | 1 | P | LSC |
| 39 | 43018 | 39 | 139672 | 1 | P | LSC/IR |
| 32 | 1312 | 32 | 113090 | 1 | P | LSC/SSC |
| 30 | 66915 | 30 | 113994 | 1 | P | LSC/SSC |
| 35 | 66932 | 35 | 121330 | 1 | P | LSC/SSC |
| 31 | 66936 | 31 | 121332 | 1 | P | LSC/SSC |
| 31 | 70842 | 31 | 121318 | 1 | P | LSC/SSC |
| 30 | 70843 | 30 | 121316 | 1 | P | LSC/SSC |
| 32 | 70846 | 32 | 121315 | 1 | P | LSC/SSC |
| 34 | 1295 | 34 | 66934 | 1 | R | LSC |
| 30 | 1316 | 30 | 113089 | 1 | R | LSC/SSC |
| 30 | 3520 | 30 | 113070 | 1 | R | LSC/SSC |
| 36 | 66931 | 36 | 121331 | 1 | R | LSC/SSC |
| 30 | 113098 | 30 | 124979 | 1 | R | LSC/SSC |

Table S3 Tandem repeat sequences in the *Platanthera ussuriensis* chloroplast genome.

| Number | Indices | Repeat lenth (bp) | Copy number | Repeat unit | Location | Region |
| --- | --- | --- | --- | --- | --- | --- |
| 1 | 1297--1344 | 14 | 3 | ATATAAATATATAT | IGS (*psbA*, *trnK-UUU*) | LSC |
| 2 | 1297--1356 | 10 | 6 | ATATATATAT | IGS (*psbA*, *trnK-UUU*) | LSC |
| 3 | 1290--1351 | 21 | 3 | ATAAAATATATATATATATAT | IGS (*psbA*, *trnK-UUU*) | LSC |
| 4 | 3289--3332 | 20 | 2 | TATGTGTATATTTTATATCT | intron (*trnK-UUU*) | LSC |
| 5 | 28489--28592 | 7 | 13 | TATATTA | IGS (*petN*, *psbM*) | LSC |
| 6 | 28511--28592 | 21 | 4 | TAGTATTATATTATATAGTTA | IGS (*petN*, *psbM*) | LSC |
| 7 | 28511--28581 | 29 | 2 | TAGTATATAGTATATATATTATAGTATTA | IGS (*petN*, *psbM*) | LSC |
| 8 | 41513--41560 | 25 | 2 | AAAATCTAATAGATAATATAAAAAT | IGS (*psaA*, *ycf3*) | LSC |
| 9 | 47032--47063 | 7 | 5 | CTATTAT | intron (*trnL-UAA*) | LSC |
| 10 | 47059--47099 | 11 | 4 | TATTAGTAGTA | intron (*trnL-UAA*) | LSC |
| 11 | 47131--47156 | 11 | 2 | CTATTTATATT | intron (*trnL-UAA*) | LSC |
| 12 | 51969--51996 | 14 | 2 | TTTGATTACTACTA | IGS (*trnM-CAU*, *atpE*) | LSC |
| 13 | 65562--65597 | 13 | 3 | TATTAACTATATA | IGS (*trnP-UGG*, *psaJ*) | LSC |
| 14 | 66905--66958 | 17 | 3 | TATTTTATATATTAATA | IGS (*rp133*, *rps18*) | LSC |
| 15 | 66894--66942 | 24 | 2 | ATTTTCTATATATTAATATATTAA | IGS (*rp133*, *rps18*) | LSC |
| 16 | 66909--66966 | 15 | 4 | TTATATATTAATATA | IGS (*rp133*, *rps18*) | LSC |
| 17 | 66935--66959 | 11 | 2 | ATATATTATAT | IGS (*rp133*, *rps18*) | LSC |
| 18 | 70720--70762 | 21 | 2 | TAATATATTCATATAATATAT | intron (*clpP*) | LSC |
| 19 | 91856--91915 | 24 | 3 | TGATGATAGTGACGATACCAATAT | *ycf2* | IR |
| 20 | 113954--113984 | 15 | 2 | TGTAATATTAAATAT | IGS (*rp132*, *trnL-UAG*) | SSC |
| 21 | 113991--114069 | 26 | 3 | ATTAAATAATATTTAATATAAAAAAA | IGS (*rp132*, *trnL-UAG*) | SSC |
| 22 | 121330--121363 | 11 | 3 | ATATATATTAT | intron (*ndhA*) | SSC |
| 23 | 125389--125428 | 21 | 2 | AAATTGGTATTCCTATCAACA | *yfc1* | SSC |
| 24 | 147086--147145 | 24 | 3 | TCACTATCATCAATATAAATATCG | *ycf2* | IR |

IGS represents intergenic spacer.

Table S4 Total number of SSRs identified within the chloroplast genome of *Platanthera ussuriensis*.

| Repeats | 3 | 4 | 5 | 6 | 7 | 8 | 9 | 10 | 11 | 12 | 13 | 14 | 15 | 16 | 17 | 18 | 19 | 20 | 21 | 22 | 23 | Total |
| --- | --- | --- | --- | --- | --- | --- | --- | --- | --- | --- | --- | --- | --- | --- | --- | --- | --- | --- | --- | --- | --- | --- |
| A/T | - | - | - | - | - | - | - | 22 | 17 | 11 | 5 | 3 | 2 | 1 | 1 | 1 | 1 | - | 2 | - | 1 | 67 |
| C/G | - | - | - | - | - | - | - | 1 | - | - | - | - | - | - | - | - | - | - | - | - | - | 1 |
| AG/CT | - | - | 2 | - | - | - | - | - | - | - | - | - | - | - | - | - | - | - | - | - | - | 2 |
| AT/AT | - | - | 8 | 6 | 2 | 3 | 2 | - | - | - | - | - | - | - | - | - | - | - | - | - | - | 21 |
| AAT/ATT | - | 7 | - | - | - | - | - | - | - | - | - | - | - | - | - | - | - | - | - | - | - | 7 |
| AGG/CCT | - | 1 | - | - | - | - | - | - | - | - | - | - | - | - | - | - | - | - | - | - | - | 1 |
| AAAT/ATTT | 2 | - | - | - | - | - | - | - | - | - | - | - | - | - | - | - | - | - | - | - | - | 2 |
| AATG/ATTC | 1 | - | - | - | - | - | - | - | - | - | - | - | - | - | - | - | - | - | - | - | - | 1 |
| ACAG/CTGT | 1 | - | - | - | - | - | - | - | - | - | - | - | - | - | - | - | - | - | - | - | - | 1 |
| AGAT/ATCT | 1 | - | - | - | - | - | - | - | - | - | - | - | - | - | - | - | - | - | - | - | - | 1 |
| Total |  |  |  |  |  |  |  |  |  |  |  |  |  |  |  |  |  |  |  |  |  | 104 |

Table S5 SSRs within the *Platanthera ussuriensis* chloroplast genome.

| No. | SSR type | Size | Region | Start | End |
| --- | --- | --- | --- | --- | --- |
| 1 | (AT)8 | 16 | LSC | 1,297 | 1,312 |
| 2 | (TA)6 | 12 | LSC | 1,324 | 1,335 |
| 3 | (TA)8 | 16 | LSC | 3,504 | 3,519 |
| 4 | (TA)7 | 14 | LSC | 3,532 | 3,545 |
| 5 | (A)10 | 10 | LSC | 4,421 | 4,430 |
| 6 | (AAT)4 | 12 | LSC | 4,429 | 4,440 |
| 7 | (T)10 | 10 | LSC | 4,563 | 4,572 |
| 8-9 | (C)10(A)10 | 20 | LSC | 4,872 | 4,891 |
| 10 | (AT)5 | 10 | LSC | 5,880 | 5,889 |
| 11 | (AT)6 | 12 | LSC | 6,388 | 6,399 |
| 12 | (T)10 | 10 | LSC | 6,399 | 6,408 |
| 13 | (A)11 | 11 | LSC | 6,921 | 6,931 |
| 14 | (TA)5 | 10 | LSC | 7,486 | 7,495 |
| 15 | (A)11 | 11 | LSC | 7,590 | 7,600 |
| 16 | (A)11 | 11 | LSC | 7,671 | 7,681 |
| 17 | (TA)5 | 10 | LSC | 8,352 | 8,361 |
| 18 | (T)10 | 10 | LSC | 8,539 | 8,548 |
| 19 | (T)10 | 10 | LSC | 8,747 | 8,756 |
| 20 | (A)13 | 13 | LSC | 8,870 | 8,882 |
| 21 | (TCTA)3 | 12 | LSC | 9,626 | 9,637 |
| 22 | (GTCT)3 | 12 | LSC | 10,761 | 10,772 |
| 23 | (A)12 | 12 | LSC | 12,064 | 12,075 |
| 24 | (AT)6 | 12 | LSC | 13,500 | 13,511 |
| 25 | (T)10 | 10 | LSC | 15,448 | 15,457 |
| 26 | (T)10 | 10 | LSC | 17,487 | 17,496 |
| 27 | (T)10 | 10 | LSC | 17,554 | 17,563 |
| 28 | (T)11 | 11 | LSC | 17,660 | 17,670 |
| 29 | (AT)5 | 10 | LSC | 19,048 | 19,057 |
| 30 | (T)12 | 12 | LSC | 19,987 | 19,998 |
| 31 | (A)11 | 11 | LSC | 27,155 | 27,165 |
| 32 | (AAT)4 | 12 | LSC | 27,164 | 27,175 |
| 33 | (A)21 | 21 | LSC | 28,440 | 28,460 |
| 34 | (TA)7 | 14 | LSC | 28,514 | 28,527 |
| 35 | (TA)6 | 12 | LSC | 28,897 | 28,908 |
| 36 | (A)10 | 10 | LSC | 29,667 | 29,676 |
| 37 | (T)10 | 10 | LSC | 30,681 | 30,690 |
| 38 | (TA)5 | 10 | LSC | 30,752 | 30,761 |
| 39 | (A)13 | 13 | LSC | 31,044 | 31,056 |
| 40 | (AT)5 | 10 | LSC | 31,313 | 31,322 |
| 41 | (A)12 | 12 | LSC | 31,512 | 31,523 |
| 42 | (ATA)4 | 12 | LSC | 35,561 | 35,572 |
| 43 | (T)12 | 12 | LSC | 41,428 | 41,439 |
| 44 | (T)10 | 10 | LSC | 43,610 | 43,619 |
| 45 | (A)13 | 13 | LSC | 45,961 | 45,973 |
| 46 | (T)23 | 23 | LSC | 46,088 | 46,110 |
| 47 | (A)11 | 11 | LSC | 46,819 | 46,829 |
| 48 | (T)21 | 21 | LSC | 50,361 | 50,381 |
| 49 | (A)11 | 11 | LSC | 50,894 | 50,904 |
| 50 | (A)11 | 11 | LSC | 51,478 | 51,488 |
| 51 | (A)12 | 12 | LSC | 56,761 | 56,772 |
| 52 | (A)11 | 11 | LSC | 58,302 | 58,312 |
| 53 | (AT)6 | 12 | LSC | 59,220 | 59,231 |
| 54 | (AATG)3 | 12 | LSC | 61,250 | 61,261 |
| 55 | (AGG)4 | 12 | LSC | 65,078 | 65,089 |
| 56 | (T)12 | 12 | LSC | 65,531 | 65,542 |
| 57 | (A)17 | 17 | LSC | 66,509 | 66,525 |
| 58 | (A)15 | 15 | LSC | 66,870 | 66,884 |
| 59 | (TA)5 | 10 | LSC | 66,941 | 66,950 |
| 60 | (T)14 | 14 | LSC | 67,467 | 67,480 |
| 61 | (T)10 | 10 | LSC | 68,694 | 68,703 |
| 62 | (A)14 | 14 | LSC | 69,416 | 69,429 |
| 63 | (AATA)3 | 12 | LSC | 69,428 | 69,439 |
| 64 | (T)10 | 10 | LSC | 69,883 | 69,892 |
| 65 | (T)12 | 12 | LSC | 70,579 | 70,590 |
| 66-67 | (TA)9(T)10 | 28 | LSC | 70,844 | 70,871 |
| 68 | (A)19 | 19 | LSC | 73,637 | 73,655 |
| 69 | (T)13 | 13 | LSC | 74,188 | 74,200 |
| 70 | (T)18 | 18 | LSC | 79,598 | 79,615 |
| 71 | (A)11 | 11 | LSC | 79,620 | 79,630 |
| 72 | (TA)9 | 18 | LSC | 81,726 | 81,743 |
| 73 | (A)13 | 13 | LSC | 82,032 | 82,044 |
| 74 | (A)10 | 10 | LSC | 82,849 | 82,858 |
| 75 | (ATT)4 | 12 | LSC | 82,926 | 82,937 |
| 76 | (T)10 | 10 | LSC | 83,437 | 83,446 |
| 77 | (T)11 | 11 | IRb | 84,514 | 84,524 |
| 78 | (GA)5 | 10 | IRb | 90,072 | 90,081 |
| 79 | (A)16 | 16 | SSC | 112,780 | 112,795 |
| 80 | (A)11 | 11 | SSC | 112,963 | 112,973 |
| 81 | (TA)6 | 12 | SSC | 113,100 | 113,111 |
| 82 | (T)12 | 12 | SSC | 113,119 | 113,130 |
| 83 | (TTA)4 | 12 | SSC | 113,746 | 113,757 |
| 84 | (A)11 | 11 | SSC | 114,114 | 114,124 |
| 85 | (TAT)4 | 12 | SSC | 114,256 | 114,267 |
| 86 | (ATA)4 | 12 | SSC | 114,452 | 114,463 |
| 87 | (T)12 | 12 | SSC | 115,059 | 115,070 |
| 88 | (TTTA)3 | 12 | SSC | 117,920 | 117,931 |
| 89 | (T)14 | 14 | SSC | 119,775 | 119,788 |
| 90 | (A)12 | 12 | SSC | 121,246 | 121,257 |
| 91-92 | (A)10(TA)8 | 26 | SSC | 121,321 | 121,346 |
| 93 | (TA)5 | 10 | SSC | 121,348 | 121,357 |
| 94 | (T)12 | 12 | SSC | 124,980 | 124,991 |
| 95 | (A)10 | 10 | SSC | 126,102 | 126,111 |
| 96 | (T)10 | 10 | SSC | 126,605 | 126,614 |
| 97 | (T)11 | 11 | SSC | 126,764 | 126,774 |
| 98 | (T)11 | 11 | SSC | 126,784 | 126,794 |
| 99 | (A)10 | 10 | SSC | 126,816 | 126,825 |
| 100 | (T)15 | 15 | SSC | 128,055 | 128,069 |
| 101 | (T)10 | 10 | SSC | 128,176 | 128,185 |
| 102 | (A)11 | 11 | SSC | 128,331 | 128,341 |
| 103 | (TC)5 | 10 | IRa | 148,920 | 148,929 |
| 104 | (A)11 | 11 | IRa | 154,477 | 154,487 |

Table S6 RNA editing sites and amino acid changes in the chloroplast genome of *Platanthera ussuriensis*.

| Gene | Nt Pos | AA Pos | Align Col | Codon change | Amino acid change | Score |
| --- | --- | --- | --- | --- | --- | --- |
| *accD* | 1139 | 380 | 423 | TCA => TTA | S => L | 1.00 |
|  | 1261 | 421 | 464 | CAC => TAC | H => Y | 1.00 |
|  | 1343 | 448 | 491 | TCA => TTA | S => L | 1.00 |
|  | 1385 | 462 | 505 | CCT => CTT | P => L | 1.00 |
| *atpA* | 773 | 258 | 258 | TCA => TTA | S => L | 1.00 |
|  | 914 | 305 | 305 | TCA => TTA | S => L | 1.00 |
|  | 1148 | 383 | 383 | TCA => TTA | S => L | 1.00 |
| *atpB* | 1184 | 395 | 395 | TCA => TTA | S => L | 1.00 |
| *atpF* | 92 | 31 | 31 | CCA => CTA | P => L | 0.86 |
| *atpI* | 629 | 210 | 213 | TCA => TTA | S => L | 1.00 |
| *ccsA* | 376 | 126 | 129 | CCC => TCC | P => S | 0.86 |
| *clpP* | 82 | 28 | 28 | CAT => TAT | H => Y | 1.00 |
|  | 559 | 187 | 187 | CAT => TAT | H => Y | 1.00 |
| *matK* | 85 | 29 | 29 | CTT => TTT | L => F | 1.00 |
|  | 490 | 164 | 170 | CAT => TAT | H => Y | 1.00 |
|  | 931 | 311 | 317 | CAT => TAT | H => Y | 1.00 |
|  | 1279 | 427 | 433 | CAC => TAC | H => Y | 1.00 |
| *ndhA* | 476 | 159 | 159 | TCA => TTA | S => L | 1.00 |
|  | 1073 | 358 | 358 | TCT => TTT | S => F | 1.00 |
| *ndhB* | 149 | 50 | 50 | TCA => TTA | S => L | 1.00 |
|  | 467 | 156 | 156 | CCA => CTA | P => L | 1.00 |
|  | 586 | 196 | 196 | CAT => TAT | H => Y | 1.00 |
|  | 704 | 235 | 235 | TCC => TTC | S => F | 1.00 |
|  | 737 | 246 | 246 | CCA => CTA | P => L | 1.00 |
|  | 830 | 277 | 277 | TCA => TTA | S => L | 1.00 |
|  | 836 | 279 | 279 | TCA => TTA | S => L | 1.00 |
|  | 1112 | 371 | 371 | TCA => TTA | S => L | 1.00 |
|  | 1193 | 398 | 398 | TCA => TTA | S => L | 1.00 |
|  | 1435 | 479 | 479 | CCC => TCC | P => S | 1.00 |
|  | 1481 | 494 | 494 | CCA => CTA | P => L | 1.00 |
| *ndhD* | 59 | 20 | 20 | ACG => ATG | T => M | 1.00 |
|  | 116 | 39 | 39 | TCA => TTA | S => L | 1.00 |
|  | 242 | 81 | 81 | ACC => ATC | T => I | 1.00 |
|  | 731 | 244 | 244 | TCG => TTG | S => L | 1.00 |
|  | 935 | 312 | 312 | TCA => TTA | S => L | 1.00 |
|  | 1507 | 503 | 503 | CCA => TCA | P => S | 1.00 |
| *ndhF* | 62 | 21 | 21 | TCA => TTA | S => L | 1.00 |
|  | 290 | 97 | 97 | TCA => TTA | S => L | 1.00 |
|  | 379 | 127 | 127 | CAT => TAT | H => Y | 1.00 |
|  | 578 | 193 | 193 | ACC => ATC | T => I | 1.00 |
|  | 1751 | 584 | 596 | ACA => ATA | T => I | 1.00 |
|  | 2222 | 741 | 753 | TCT => TTT | S => F | 1.00 |
| *ndhG* | 155 | 52 | 52 | TCA => TTA | S => L | 1.00 |
| *petB* | 418 | 140 | 140 | CGG => TGG | R => W | 1.00 |
|  | 611 | 204 | 204 | CCA => CTA | P => L | 1.00 |
| *petD* | - | - | - | - | - | - |
| *petG* | - | - | - | - | - | - |
| *petL* | - | - | - | - | - | - |
| *psaB* | - | - | - | - | - | - |
| *psaI* | 80 | 27 | 43 | TCT => TTT | S => F | 0.86 |
| *psbB* | - | - | - | - | - | - |
| *psbE* | - | - | - | - | - | - |
| *psbF* | 77 | 26 | 26 | TCT => TTT | S => F | 1.00 |
| *psbL* | - | - | - | - | - | - |
| *rpl2* | - | - | - | - | - | - |
| *rpl20* | 26 | 9 | 9 | ACA => ATA | T => I | 0.86 |
|  | 308 | 103 | 103 | TCA => TTA | S => L | 0.86 |
| *rpl23* | - | - | - | - | - | - |
| *rpoA* | 368 | 123 | 123 | TCA => TTA | S => L | 1.00 |
|  | 830 | 277 | 279 | TCA => TTA | S => L | 1.00 |
| *rpoB* | 338 | 113 | 113 | TCT => TTT | S => F | 1.00 |
|  | 473 | 158 | 159 | TCG => TTG | S => L | 0.86 |
|  | 551 | 184 | 185 | TCA => TTA | S => L | 1.00 |
|  | 623 | 208 | 209 | CCG => CTG | P => L | 0.86 |
|  | 1934 | 645 | 662 | GCT => GTT | A => V | 0.86 |
|  | 2426 | 809 | 827 | TCA => TTA | S => L | 0.86 |
| *rpoC1* | 62 | 21 | 21 | CCA => CTA | P => L | 1.00 |
|  | 203 | 68 | 68 | TCC => TTC | S => F | 1.00 |
|  | 278 | 93 | 93 | TCT => TTT | S => F | 1.00 |
|  | 638 | 213 | 220 | TCG => TTG | S => L | 1.00 |
|  | 808 | 270 | 286 | CGG => TGG | R => W | 1.00 |
|  | 1643 | 548 | 565 | GCA => GTA | A => V | 0.86 |
| *rpoC2* | 2311 | 771 | 955 | CGG => TGG | R => W | 1.00 |
|  | 3746 | 1249 | 1457 | TCA => TTA | S => L | 0.86 |
| *rps2* | - | - | - | - | - | - |
| *rps8* | 182 | 61 | 61 | TCA => TTA | S => L | 0.86 |
| *rps14* | 80 | 27 | 27 | TCA => TTA | S => L | 1.00 |
|  | 149 | 50 | 53 | CCA => CTA | P => L | 1.00 |
| *rps16* | 143 | 48 | 48 | TCA => TTA | S => L | 1.00 |
| *ycf3* | 44 | 15 | 15 | TCT => TTT | S => F | 1.00 |
|  | 185 | 62 | 62 | ACG => ATG | T => M | 1.00 |
|  | 191 | 64 | 64 | CCA => CTA | P => L | 1.00 |

Notes: Nt Pos - The location of the nucleotide predicted to be edited in the input DNA sequence; AA Pos - The location of the amino acid predicted to be edited in the translation of the input DNA sequence; Align Col - The column in the AA alignment where the edit site occurs.
